# Strategies to Cope with Phases of Spiritual Dryness in Seventh-Day Adventists

**DOI:** 10.1007/s10943-020-01093-6

**Published:** 2020-10-31

**Authors:** Arndt Büssing, Lorethy Starck, Klaus van Treeck

**Affiliations:** 1 Professorship Quality of Life, Spirituality and Coping, Institute of Integrative Medicine, Faculty of Health, Witten/Herdecke University, Gerhard-Kienle Weg 4, 58313 Herdecke, Germany; 2 IUNCTUS - Competence Center for Christian Spirituality, Philosophical-Theological Academy, 48149 Münster, Germany; 3 Institute for Holistic Wellbeing and Resilience, Bremen, 28215 Germany; 4 Institute of Continuing Education of Seventh-Day-Adventists (IfW), Hannover, 30519 Germany

**Keywords:** Spiritual dryness, Strategies to cope, Spirituality, Seventh-day Adventists

## Abstract

In Adventists (*n* = 603) 45% were less effective and 55% more effective in coping with phases of spiritual dryness, particularly those with leading roles in the church. Strategies such as devotion/trust in God, private prayer/meditation/scripture reading, openness, talks with close others, and self-care were most often utilized. Their ability to cope was predicted best by low Acedia, Living from the Faith, low emotional exhaustion, well-being and having a duty in the church (*R*^2^ = .24). When these phases were overcome, several perceived spiritual depth and were able to help others all the more. Helpful strategies should be made available to support persons struggling with spiritual dryness.

## Background

In the life of a religious person, perceptions of spiritual dryness will surely occur, either occasionally (as a phase) or even again and again (as states or a process); only a few state that they do not experience this at all (Büssing et al. 2016, 2017a). Spiritual struggles, desolation and experiences of dryness (“desert experience”) are recurring topics in religious literature (Plattig and Bäumer 2010; Plattig 1994; Büssing and Dienberg 2019), as well as in psychology/health research (Exline 2013; Büssing et al. 2013). Indicators of spiritual dryness are the perception that communication with God is disturbed and that prayers go ‘unanswered’, that God is distant, not listening and not caring, or of being completely abandoned by God (Büssing et al. 2013). Some theologians´ interpretations follow John of the Cross (1542–1591) who argued that the “Dark Night of the Soul” is a purification process toward a more mature relationship with God (Johannes vom Kreuz 2013; John of the Cross 1959, 2013). The ‘desert father’ Evagrius Pontikus (345–399) mentioned ‘Acedia’ as a bored negligence or “inertia of the heart” which results in emotional fatigue, negligence in prayer life, and tiring of an ascetic life with God (Pontikos 2007). Apart from a ‘purification process’ or ‘bored negligence’, such phases could either be different or even part of a depressive state, too (Ott 1982; May 2003; Plattig and Bäumer 2010; Durà-Vilà 2017). Empirical studies among Catholic pastoral workers showed that spiritual dryness can be predicted by low perception of the sacred in life and a low sense of coherence on the one hand, and depressive states and emotional exhaustion on the other (Büssing et al. 2016, 2017a). Qualitative interviews with religious brothers and sisters revealed that the causes of spiritual dryness are complex and depend on the individual and its life situation. Five main categories of causes and triggers were identified: Loss of Relationship with God, Loss of Orientation, Loss of Depth, Difficulties with the Religious Community, and Intrinsic Factors: Overload, Uncertainty, Depression (Büssing et al. 2020a).

Interestingly, when these phases were overcome, several reported greater spiritual serenity and depth and/or that they were inspired to help others all the more. Thus, it is important to cope with spiritual dryness and to overcome these phases when they are seen as a process of spiritual growth (‘transformation’) instead of an inhibitor or prevention. The crucial point seems to be whether and how persons experience such phases of doubt, insecurity and struggle, whether they are adequately supported, and whether they find strategies to cope. In Catholic priests we identified several strategies, i.e., Explicit spiritual practices (i.e., personal prayer, meditation), Spiritual attitudes (i.e., trust in God, Christocentric orientation, openness to new things), Interpersonal communication (i.e., family, friends, spiritual community), Accompanied self-reflection and experience (i.e., spiritual counselor, psychotherapy), Charitable acting (i.e., helping others), Self-Care (i.e., private leisure life, holidays, sports), and Avoidance strategies (i.e., repression, inner retirement, distraction) (Büssing et al. 2017b). Some of these strategies to cope were more and some less helpful, i.e., utilization of Self-Care/Interpersonal Communication and/or Spirituality were more helpful to cope with spiritual dryness, while Avoidance strategies were less effective (Büssing et al. 2017b).

Spiritual dryness is not exclusive to Catholics or ancient saints (Büssing and Dienberg 2019); religious doubting and struggles may occur in different denominations, in Catholics, Protestants, and Seventh-day Adventists, although there might be differences (Kooistra and Pargament 1999; Braam et al. 2008, Büssing et al. 2020b). Spiritual dryness was observed also in Seventh-day Adventists (SDA) (Büssing et al. 2020b), who are recognized by their rather strict religious life and beneficial health behavior (Orlich et al. 2013; Morton et al. 2017). Nevertheless, also Ellen G. White (1827–1915), one of the co-founders of the SDA church in the USA, described struggles in prayer life, to getting in resonance with God and persons around her, and symptoms of spiritual/emotional exhaustion (White 1868; Vine 2014, p. 48).

In this study, we intended to analyze (1) whether SDAs found ways to cope with phases of spiritual dryness, (2) which strategies to cope were utilized, and (3) what they perceived in terms of spiritual depth and helping others after overcoming these phases. As influencing variables we will address also Acedia symptoms as described above, perception of the sacred and living from the faith as spiritual resources, low well-being and emotional exhaustion as negative influences and outcomes. As coping strategies we referred to the main topics identified in the statements of Catholic priests (Büssing et al. 2017b).

## Materials and Methods

### Study Participants

Members of the SDA Church Inter-European Division (Bern, Switzerland) were invited by email to the European coordinators, regional groups, Facebook groups, and SDA information journals to participate in an anonymous online survey with standardized measures. By filling in the anonymous questionnaire, they consented to participate. Neither concrete identifying personal details nor IP addresses were recorded to guarantee anonymity.

### Measures

The questionnaire asks for gender, age, position within the church, how long one is member of the SDA. Further variables will be described in the following.

#### Spiritual Dryness

Whether and how often phases of spiritual dryness were experienced was assessed with the 6-item Spiritual Dryness Scale (SDS) which has good internal consistency (Cronbach’s *α* = 0.87) (Büssing et al. 2013). Specific statements refer to the feelings that God is distant, that one’s prayers go unanswered, of being “spiritually empty” or not being able to give any more (in terms of spiritual exhaustion), and, finally, feelings of being abandoned by God. The items of this instrument were formulated in order to fit into the daily life experiences of religious individuals. Response options on a Likert scale were *not at all* (1), *rarely* (2), *occasionally* (3), *fairly often* (4), and *regularly* (5). The SDS scores are mean scores and represent the perceived lack/shortage.

Four additional SDS items ask for their “longing for God”, whether or not one has found ways to cope with these phases, and further perceptions and reactions when these phases were overcome, i.e., inspiration to help others all the more or greater spiritual serenity and depth. According to the response pattern of the putative more or less effective coping persons two groups were differentiated, the less effective coper (who stated that they either were *not at all*, *rarely* or *sometimes only* able to cope with spiritual dryness; 45%) and the more effective coper (who *often* or even *regularly* found ways to cope; 55%).

#### Strategies to Cope with Phases of Spiritual Dryness

Referring to the categories identified in the free text statements of Catholic priests about which strategies they used to cope with phases of spiritual dryness (Büssing et al. 2017b), ten coping strategies were formulated and the respective behaviors quantified. Response options ranged from 0 to 5 (*does not apply at all, does not really apply, half and half (neither yes nor no), applies quite a bit, applies very much*). The participants also had the option to add other useful strategies (free text format).

#### Acedia Symptoms

To measure symptoms related to Acedia in a wider context, we used the 8-item Acedia questionnaire which addresses experiences of difficulties in prayer life (in terms of inattentiveness and distance) and excessive spiritual demands (in terms of perceived overcharging demands referred to God) (Büssing et al. 2020b). Examples of the former topic are “I am more passive in prayer and without any inner involvement”, “My prayer life doesn’t excite me so much anymore”, “I really enjoy only a little in my spiritual life”, “I don’t really care whether I find God in prayer or not”, while the latter topic was addressed by items such as “What God asks of me is more than I can give”, “What God asked of me is just too much”, “I really don´t know what God wants from me”, “Somehow, everything got too much for me”. Internal consistency of the Acedia scale is good (Cronbach’s alpha = .84); the 4-item subscale Excessive Spiritual Demands has good (Cronbach’s alpha = .81) and the 4-item subscale Difficulties in Prayer Life have acceptable to good internal consistency (Cronbach´s alpha = .75). Response options on a Likert scale were *not at all* (1), *rarely* (2), *occasionally* (3), *fairly often* (4), and *regularly* (5).

#### Living from the Faith

To address whether the participants live from their faith, a subscale of the Franciscan-inspired Spirituality (FraSpir) questionnaire was used (Büssing et al. 2017c). This 13-item subscale termed *Live from the Faith*/*Search for God* has very good internal consistency (Cronbach’s alpha = 0.97). Specific items are “My faith is my orientation in life”, “My faith/spirituality gives meaning to my life”, “I have a sense of the Sacred in my life”, “I listen to God´s word in me”, “I keep times of silence before God”, “I feel a longing for nearness to God”. Items were scored on a 5-point scale from disagreement to agreement (0—*does not apply at all*; 1—*does not truly apply*; 2—*half and half* (*neither yes nor no*); 3—*applies quite a bit*; 4—*applies very much*).

#### Perception of the Sacred

Perception of the Sacred in daily life was measured with the Daily Spiritual Experience Scale in its 6-item version (DSES-6; Cronbach’s alpha = .91) (Underwood and Teresi 2002; Underwood 2011). It addresses feelings of God´s presence, God´s love, desire to be closer to God (union), finding strength/comfort in God, being touched by the beauty of creation. The items are scored from 1 to 6 (*many times a day, every day, most days, some days, once in a while* and *never/almost never*). Item scores were finally summed up.

#### Spiritual Practices and Behaviors

Frequency of spontaneous prayer (‘privately‘; apart from public worship/church service etc.) was measured with a 5-grade scaling (*several times per day*; *daily*; *on several days*; *on some days*; *rarely*/*nearly never*), while the frequency of church attendance was measured with a 4-grade scale (*at least once per week*; *several times per month*; *once per month*; *less than once per month*).

#### Well-Being

Well-being was assessed with the 5-item WHO-Five Well-being Index (WHO-5) (Bech et al. 2003). Representative items are “I have felt cheerful and in good spirits” or “My daily life has been filled with things that interest me”. The intensity of feelings within the last 2 weeks was scored from *at no time* (0) to *all the time* (5). Here, the sum scores ranging from 0 to 25 were reported. Scores < 13 may indicate depressive states.

#### Under Pressure and Emotional Exhaustion

Perceived emotional exhaustion and feelings of being ‘under pressure’ (either by stress, anxiety, etc.) were measured with two visual analog scales (VAS) ranging from *not at all* (0) to *extremely* (100).

### Statistical Analyses

Descriptive statistics, cross-tabulation (Chi^2^ test), analyses of variance (ANOVA), first order correlations (Spearman rho) and regression analyses were computed with SPSS 23.0.

Given the exploratory character of this study, the significance level of ANOVA and correlation analyses were set at *p* < 0.05. With respect to classifying the strength of the observed correlations, we regarded *r* > .5 as a strong correlation, an *r* between .3 and .5 as a moderate correlation, an *r* between .2 and .3 as a weak correlation, and *r* < .2 as negligible or no correlation.

## Results

### Study Participants

We enrolled 603 participants from Germany, Austria and the Francophone countries France, Belgium and Switzerland who have responded to the coping strategies items (Table 1). The proportion of women and men is quite balanced (45% and 55%); their mean age is 50.1 ± 15.1 years. Among them, 33% have leading roles in the church (pastor, elder, deacon), 45% other duties and 22% no duties in the church. SDA in leading roles are significantly older than those with other duties (52.9 ± 13.3 vs 48.4 ± 14.9; *p* = .002, Mann–Whitney-*U* test) and in trend also compared to those without duties (52.9 ± 13.3 vs 49.0 ± 17.0; *p* = .077). However, the age is not significantly different when those without any duties were compared with those who have a duty in the church (49.0 ± 17.0 vs 50.2 ± 14,4; n.s., Mann–Whitney-*U* test).

**Table 1:** Description of the sample (*N* = 603)

Gender (%)	
Women	45
Men	55
Age (years)	50.1 ± 15.1 [14–99]
Age groups (%)	
< 40 years	28
41–60 years	48
> 60 years	24
Country (%)	
Germany	54
Austria	27
France, Belgium and Luxembourg	19
SDA since … (years)	28.8 ± 15.8 [0–74]
Duty within the church (%)	
Pastor	14
Elder	11
Deacon	8
Other duties	45
None duty	22
How often do you pray privately? (%)	
Several times per day	57
Daily	27
At several days	9
At some days	5
Seldom/rather never	3
How often going to church? (%)	
At least once per week	72
Several times per month	18
Once per month	2
Less than once per month	8
Well-being (WHO5)	14.8 ± 5.4 [0–25]
Under pressure (VAS)	45.8 ± 28.4 [0–100]
Emotional exhaustion (VAS)	38.6 ± 30.8 [0–100]
Live from the Faith/Search for God (FraSpir)	3.0 ± 0.6 [0–4]
Perception of the Sacred (DSES-6)	4.3 ± 1.0 [1–6]
Spiritual Dryness (SDS)	2.3 ± 0.8 [0–5]

A deep longing for God was a bit higher in SDAs in leading roles (*F* = 7.03, *p* = .001), less often in persons > 60 year of age (*F* = 3.76, *p* = .024), and in trend by women (*F* = 3.61, *p* = .058) (data not shown).

SDAs´ well-being is in the lower midrange; feelings of being under pressure and perception of emotional exhaustion are a moderate range, however, with strong variance (indicating that several of them do experience burdening situations).

### Finding Ways to Cope with Phases of Spiritual Dryness

15% stated that they did not or only rarely find ways to cope with phases of spiritual dryness and 30% sometimes (these were regarded as less effective copers), 35% often and 19% regularly (they were regarded as effective copers).

Here SDAs with leading roles in the church were significantly more able to find strategies than those without any duty (Table 2). This ability was not gender related (*F* = 0.93, n.s.), but lowest in younger persons (< 40 years) (*F* = 3.08, *p* = .047).

**Table 2:** Ability to cope with spiritual dryness and reactions after

	Spiritual dryness (mean score)	Deep longing for God (mean score)	Found ways to cope with spiritual dryness (mean score)	These phases inspire to help others more (mean score)	After these phases: deeper Inner Clarity and Depth (mean score)
All persons (*n* = 600)					
*M*	2.28	3.97	3.54	3.02	3.18
SD	0.81	1.00	1.05	1.24	1.20
Duty in the church					
Leading roles (*n* = 198)					
*M*	2.08	4.15	3.80	3.24	3.36
SD	0.71	1.00	0.89	1.24	1.24
Other duties (*n* = 271)					
*M*	2.29	3.95	3.52	3.05	3.22
SD	0.79	0.96	1.06	1.21	1.19
No duty (*n* = 131)					
*M*	2.59	3.73	3.17	2.62	2.78
SD	0.92	1.102	1.15	1.20	1.08
* F* value	16.09	7.03	13.65	9.22	8.46
* p* value	< .0001	.001	< .0001	< .0001	< .0001
Finding ways to cope					
Less effective coper (not at all/rarely/sometimes) (*n* = 246)					
*M*	2.66	3.76	2.57	2.50	2.83
SD	0.81	1.02	0.68	1.10	1.12
Effective coper (often/regularly) (*n* = 302)					
*M*	2.05	4.18	4.34	3.44	3.47
SD	0.70	0.90	0.47	1.18	1.20
* F* value	87.17	26.62	1278.01	88.81	38.95
* p* value	< .0001	< .0001	< .0001	< .0001	< .0001

A deep longing for God was perceived by all persons, a bit more strongly in SDA in leading roles, in the more effective copper (Table 2), and less often in the > 60 year old persons ((*F* = 3.76, *p* = .024), and in trend by women (*F* = 3.61, *p* = .058)

### Perceptions When Phases of Spiritual Dryness were Overcome

After these phases, 12% reported that these feelings inspired them regularly to help others, 27% often, 26% sometimes, 22% rarely, and 14% not at all. Deeper spiritual clarity and depth after these phases were regularly observed by 14%, by 27% often, 32% sometimes, 14% rarely, and 12% not at all.

Those without any duty in the church had the lowest urge to help and spiritual depth after these phases compared to those with leading roles or other duties (Table 2). When the SDAs analyzed herein were effective to find ways to cope with these phases, they significantly more often experienced spiritual depth and helped others. Helping others was slightly lower in younger persons (< 40 years) compared to the other ages (*F* = 4.65, *p* = .010), while there were no age-related differences for inner depth (*F* = 0.51, n.s.). Women experienced inner depth slightly more often than men (*F* = 5.89, *p* = .016), while there were no gender-related differences for helping others after overcoming these phases (*F* = 0.00, n.s.). Therefore, it is important to analyze which strategies were used.

### Strategies to Cope with Phases of Spiritual Dryness

As shown in Table 3, the most often used strategies to cope were Devotion/Trust in God and Private prayer/Meditation/Scripture reading, followed by Openness (whatever may come), Talks with others (i.e., family. friends), and Self-Care (i.e., leisure. sport. holidays); less relevant were Avoidance strategies (i.e., suppression, inner withdrawal) and Consultation of spiritual advisor/pastor; Consultation of psychotherapists was the least relevant strategy to cope.

**Table 3:** Usage of strategies to cope with spiritual dryness

	Strategies to cope with spiritual dryness									
	Private prayer/Meditation/Scripture reading	Devotion/Trust in God	Openness (whatever may come)	Talks with others (family. friends)	Consultation of spiritual advisor/pastor	Consultation of psychotherapists	Self-Care (leisure. sport. holidays)	Spiritual retreats	Voluntary work: helping others	Avoidance strategies (suppression. inner withdrawal)
All persons (*n* = 600)										
M	3.03	3.12	2.85	2.76	1.62	0.77	2.49	1.36	2.06	1.50
SD	1.04	0.96	1.06	1.14	1.22	1.14	1.12	1.29	1.20	1.24
Duty in the church										
Leading roles (*n* = 198)										
*M*	3.24	3.28	2.91	2.77	1.70	0.70	2.59	1.68	2.21	1.39
SD	0.93	0.82	1.08	1.15	1.25	1.09	1.05	1.30	1.20	1.27
Other duties (*n* = 271)										
*M*	2.99	3.14	2.82	2.80	1.58	0.73	2.42	1.28	2.06	1.49
SD	1.01	0.95	1.06	1.13	1.19	1.16	1.15	1.31	1.19	1.20
No duty (*n* = 131)										
*M*	2.82	2.86	2.80	2.68	1.58	0.95	2.49	1.02	1.85	1.69
SD	1.19	1.12	1.02	1.17	1.24	1.17	1.13	1.11	1.23	1.27
* F* value	**6.90**	**7.60**	0.53	0.49	0.62	2.30	1.28	**11.20**	3.36	2.26
* p* value	**.001**	**.002**	n.s.	n.s.	n.s.	n.s.	n.s.	**< .0001**	.035	n.s.
Finding ways to cope										
Less effective coper (not at all/rarely/sometimes) (*n* = 246)										
*M*	2.92	2.98	2.73	2.69	1.62	0.87	2.50	1.33	1.97	1.75
SD	1.02	0.93	0.99	1.15	1.25	1.20	1.09	1.25	1.20	1.24
Effective coper (often/regularly) (*n* = 302)										
*M*	3.13	3.26	2.91	2.79	1.61	0.67	2.47	1.42	2.12	1.28
SD	1.06	0.95	1.11	1.12	1.22	1.08	1.16	1.32	1.23	1.22
* F* value	5.18	**12.85**	3.92	1.21	0.01	4.17	0.10	0.65	2.02	**19.25**
* p* value	.023	**< .0001**	.048	n.s.	n.s.	.042	n.s.	n.s.	n.s.	**< .0001**

Interestingly, phases of spiritual dryness were experienced significantly more often by persons without duties in the church as compared to those with leading roles or other duties, and they also found strategies to cope less often (Table 3). With respect to the coping strategies, the spirituality-related strategies were less often used by persons without duties, particularly Spiritual retreats, Devotion/Trust in God and Private prayer/Meditation/Scripture reading, but also Voluntary work for the benefit of others was less often utilized. All other strategies were not related to the duty in the church.

Those who stated that they have found ways to cope with spiritual dryness (often or even regularly) had significantly lower spiritual dryness cores, while those who were less able to cope (not at all, rarely or sometimes only) had the highest spiritual dryness scores (Table 3). Both coping groups showed significant differences in their use of Devotion/Trust in God, which were mainly used by those who often to regularly find ways to cope with spiritual dryness, and Avoidance strategies, which were used less often by those who regularly find ways to cope and more often by those who did not or rarely only find ways to cope (Table 3). Consultation of a psychotherapist was a little higher in the less effective spiritual dryness copers; further Private prayer/Meditation/Scripture reading and also Openness were slightly higher in the good copers compared to the poor copers.

The Consultation of psychotherapists differed between women and men, which was more often used by men than women (*F* = 8.80, *p* = .003); also Avoidance strategies were a bit more often used by men compared to women (*F* = 4.82, *p* = .028) (data not shown). All other strategies did not significantly differ between women and men. With respect to age, there was a weak difference for Self-Care, which was highest in persons < 40 years of age (*F* = 3.13, *p* = .044), while all other strategies showed no significant age-related differences (data not shown).

### Correlations Between Strategies to Cope and Indicators of Spirituality and Well-being

Some strategies were moderately to strongly interrelated (Table 4), i.e., Devotion/Trust in God were strongly related to Private prayer/Meditation/Scripture reading, and moderately with Openness. Talking with a spiritual advisor/pastor was moderately related with talking with family and friends or with a psychotherapist. Avoidance strategies were inversely and weakly related with Devotion/Trust in God. Interestingly, Avoidance was positively related to consultation with a psychotherapist, although marginally only. All other strategies were either not at all or marginally to weakly interrelated (Table 4).

**Table 4:** Correlation analyses

	Private prayer/meditation/scripture reading	Devotion/trust in God	Openness (whatever may come)	Talks with others (family. friends)	Consultation of spiritual advisor/pastor	Consultation of psychotherapists	Self-Care (leisure. sport. holidays)	Spiritual retreats	Voluntary work: helping others	Avoidance strategies (suppression. inner withdrawal)
*Strategies to cope*										
Private prayer/meditation/scripture reading	1.000									
Devotion/trust in God	.572**	1.000								
Openness (whatever may come)	.291**	.472**	1.000							
Talks with others (family. friends)	.172**	.254**	.287**	1.000						
Consultation of spiritual advisor/pastor	.173**	.158**	.196**	.421**	1.000					
Consultation of psychotherapists	− .055	− .126**	− .015	.201**	.374**	1.000				
Self-Care (leisure. sport. holidays)	.036	.067	.179**	.135**	.071	.202**	1.000			
Spiritual retreats	.189**	.054	− .042	.072	.199**	.127**	.168**	1.000		
Voluntary work: helping others	.190**	.277**	.210**	.259**	.203**	.009	.200**	.219**	1.000	
Avoidance strategies	− .181**	− .230**	− .112**	− .098	− .005	.176**	.080	− .005	− .033	1.000
Found ways to cope with spiritual dryness (SDS)	.149**	.202**	.122**	.078	− .004	− .117**	− .015	.026	.055	− .213**
*Indicators of spirituality*										
Live from the Faith/Search for God (FraSpir)	.316**	.311**	.171**	.110**	.103	− .010	.025	.208**	.145**	− .163**
Perception of the Sacred (DSES-6)	.343**	.354**	.167**	.134**	.100	− .026	.058	.199**	.240**	− .176**
Frequency of private prayer	.332**	.225**	.085*	.031	.065	− .088*	.001	.074	.070	− .151**
Frequency of attending Sunday service	.141**	.164**	.053	.071	.084*	− .116**	− .013	.057	.070	− .103*
*Spiritual struggles*										
Spiritual Dryness (DSES-6)	− .278**	− .300**	− .203**	− .122**	− .046	.114**	− .063	− .077	− .178**	.300**
Acedia Score (AcQ)	− .319**	− .301**	− .188**	− .119**	− .118**	.097	− .006	− .063	− .177**	.363**
Acedia: Excessive Spiritual Demands	− .209**	− .244**	− .182**	− .120**	− .096	.121**	− .025	− .069	− .171**	.355**
Acedia: Difficulties in Prayer Life	− .351**	− .283**	− .157**	− .093	− .120**	.035	− .012	− .054	− .126**	.285**
These phases inspire me more to help others	.201**	.206**	.099	.079	.037	− .056	.034	.205**	.292**	− .120**
After these phases greater spiritual serenity and depth	.173**	.157**	.052	.098	.060	.000	− .005	.186**	.107	− .091
*Well-being*										
Under pressure (VAS)	− .093	− .154**	− .136**	− .106	− .045	.086	− .017	− .059	− .105	.244**
Emotional exhaustion (VAS)	− .144**	− .206**	− .203**	− .120**	− .047	.143**	.002	− .022	− .079	.288**
Well-being (WHO5)	.192**	.239**	.174**	.117**	.036	− .118**	.049	.122**	.153**	− .250**

**p < .001 (Spearman rho); moderate to strong correlations were highlighted (bold)

Both spirituality-related strategies (Private prayer/Meditation/Scripture reading and Devotion/Trust in God) were moderately related to Living from the Faith, Perception of the Sacred and inversely with Spiritual Dryness and Acedia (Table 4). However, Private prayer/Meditation/Scripture reading as a strategy was marginally only associated with well-being and lower emotional exhaustion, while Devotion/Trust in God was at least weakly related with both well-being indicators. All other strategies were not at all or only marginally related to indicators of spirituality or well-being. In contrast, Avoidance strategies were not relevantly related to Living from Faith or Perception of the Sacred, but marginally positive with Spiritual Dryness and Acedia, and weakly with reduced well-being, emotional exhaustion and feelings of pressure (Table 4).

Will SDAs´ religious practices have an effect on the utilized coping strategies? Frequency of private prayer was moderately related to the coping strategies Private prayer/Meditation/Scripture reading, and weakly with Devotion/Trust in God, but was not relevantly related to the other strategies, while frequency of attending the Sabbath service was marginally only related with spiritual coping strategies (Table 4).

With respect to the reactions when phases of spiritual dryness were overcome, it was found that none of the coping strategies showed relevant associations with the perception of greater spiritual serenity and depth, while the inspiration to help others more was weakly associated with concrete Voluntary work for others as a strategy, and also with Private prayer/Meditation/Scripture reading, Devotion/Trust in God and participating in Spiritual retreats (Table 4).

### Predictors of Spiritual Dryness and Finding Ways to Cope

The aforementioned strategies are the reactions toward the experience of spiritual dryness. Whether these phases were in fact prevented by the respective usage cannot be answered. Instead, one can ask whether these coping strategies may buffer the perception of spiritual dryness, and furthermore which variables could predict whether the persons have found ways to cope with spiritual dryness or not. For that purpose stepwise regression analyses were performed.

It was found that spiritual dryness was predicted by four strategies: Avoidance (Beta = .26, *T* = 6.26, *p* < .0001), low Private prayer/Meditation/Scripture reading (Beta = − .16, *T* = -3.30, *p* = .001), low Voluntary work: helping others (Beta = − .11, *T* = − 2.63, *p* = .009), and low Devotion/Trust in God (Beta = − .12, *T* = 2.46, *p* = .014). This model explains 18% of variance only (Avoidance strategy alone would predict 10% of variance).

The ability to find ways to cope with spiritual dryness was predicted (“buffered”) by five variables: low Acedia (Beta = − .12, *T* = − 2.30, *p* = .022), Living from the Faith (Beta = .22, *T* = 4.86, *p* < .0001), low emotional exhaustion (Beta = − .17, *T* = − 3.20, *p* = .001), well-being (Beta = .12, *T* = 2.22, *p* = .027) and by duties in the church (Beta = .09, *T* = 2.12, *p* = .034). This model explains 24% of variance (14% by Acedia alone). Private prayer/Meditation/Scripture reading, Devotion/Trust in God, Avoidance strategies, Perception of the Sacred and age had no significant independent influence in this model.

### Distribution of Spiritual Dryness Cluster Types and Effectiveness of Coping with Spiritual Dryness

Based on the categorical variable spiritual dryness and the continual variables well-being (WHO5), Perception of the Sacred (DSES-6), Acedia´s Excessive Spiritual Demands, Acedia´ Difficulties in Prayer Life and emotional exhaustion (VAS) one may find three cluster of SDAs´ spiritual dryness experience (Büssing et al. 2020b). As shown in Table 5, there is a clear separation of Cluster types and their ability to find ways to cope with spiritual dryness. Persons in Cluster 1 (high spiritual dryness and Acedia, low perception of the sacred, low well-being and high emotional exhaustion) have a higher proportion of less effective copers, while persons in the contrasting Cluster 3 (low spiritual dryness and low acedia, high perception of the sacred, high well-being and low emotional exhaustion) have a high proportion of effective copers. SDAs in Cluster 2 (which is a moderate intermix of the characteristics of Clusters 1 and 3) have a higher proportion of effective copers, but less clear-cut compared to Cluster 3 SDAs.

**Table 5:** Distribution of spiritual dryness cluster types and effectiveness of coping with these phases

	Found ways to cope with spiritual dryness		All coping persons
	less effective coper (not at all/rarely/sometimes)	Effective coper (often/regularly)	
Cluster 1			
*n*	121	56	177
%	68.4	31.6	100.0
Cluster 2			
*n*	70	103	173
%	40.5	59.5	100.0
Cluster 3			
*n*	47	135	182
%	25.8	74.2	100.0
All cluster			
*n*	238	294	532
%	44.7	55.3	100.0

*p* < .0001 (Pearson’s Chi^2^)

Within these three clusters, analyses of variance revealed significant differences in mean values of Avoidance strategies (*F* = 31.53, *p* < 0.0001), Private prayer/Meditation/Scripture reading (*F* = 18.39, *p* < .0001), Devotion/Trust in God (*F* = 15.79, *p* < .0001), Voluntary work: helping others (*F* = 8.72, *p* < 0.0001), Openness (whatever may come) (*F* = 6.65, *p* = 0.001), and Consultation of psychotherapists (*F* = 4.16, *p* = .016), which were lowest in persons of Cluster 1 (data not shown).

## Discussion

This study showed that the abilities to cope with phases of spiritual dryness are heterogeneous. One may find 45% less effective and 55% more effective coping persons in the sample of SDAs. Particularly the younger persons and those without any duties in the church were less effective in coping than older persons and those with duties. These differences cannot solely be attributed to differences in age, which was not significantly different when those without any duties were compared with those who have a duty in the church. Involvement in the processes of the church and taking on responsibilities might be crucial to feel accepted and involved, which may have an influence on how they can cope with spiritual struggles.

With respect to the utilized strategies to cope with spiritual dryness, spirituality-related strategies (Private prayer/Meditation/Scripture reading and Devotion/Trust in God) were used most frequently, talks with family and friends occurred more often than talks with spiritual advisor or pastor. When such phases were regarded as a matter of failure, guilt or ‘weakness in faith’ by the individual, one surely will first use strategies of the ‘inner circle’ of resources (intrinsic resources such as spirituality and self-care strategies, and also friends and family as close external resources) instead of pastors (more distant external resource) who might be more judgmental. Also self-care strategies (i.e., leisure, sport and holidays as internal resources) were of relevance, while avoidance strategies and spiritual retreats were less often used, and a consultation of a psychotherapist (as a more distant external resource) rare.

Persons without a duty in the church used Spiritual retreats, Private prayer/Meditation/Scripture reading and Devotion/Trust in God significantly less often, while neither the communication related strategies, Self-care strategies nor Avoidance strategies were used differently in the respective duty groups. It seems that their spiritual trust or endurance might be a crucial point to more effectively cope with spiritual dryness. Indeed, the less effective copers were significantly less often using Devotion/Trust in God and more often Avoidance strategies compared to the more effective coping persons. These differences cannot be explained by age or gender-related differences, and seem to be associated with other behaviors.

Although the correlation pattern of spirituality-related coping strategies and Avoidance strategies is different, there are no simple explanations. Avoidance strategies might be less meaningful to deal with spiritual dryness when it is seen as a process of spiritual growth (which would then be slowed down), while it might be relevant when one would interpret spiritual dryness as a matter of weakness and failure (which would thus be prevented). These Avoidance strategies were in fact moderately related with emotional exhaustion, feelings of being under pressure and low well-being on the one hand, and spiritual dryness and Acedia on the other hand, which would thus indicate that it is a reaction toward a spiritual burden that affects a person´s well-being. In contrast, the spirituality-related strategies were more related with living from the faith and perception of the Sacred in life, low spiritual dryness and low Acedia, and only weakly with positive well-being and low emotional exhaustion. This would indicate that Private prayer/Meditation/Scripture reading and Devotion/Trust in God are utilized because of a vital faith—despite the experience of spiritual dryness. In contrast, when Avoidance strategies are used, it does not mean one has lost faith; nevertheless, one cannot ignore the negative association, yet it is only marginal to weak. Avoidance strategies might not be dysfunctional in general. In Catholic priests it was found that Avoidance combined with other coping strategies was quite as effective as spirituality-related strategies alone (Büssing et al. 2017b). It is important that it is not the sole strategy but that one has access to other resources, too.

In this study, we can state only which strategies were used in response to phases of spiritual dryness, and which strategies were perceived as helpful. It is likely that these strategies were differentially combined, as indicated by the moderate to strong intercorrelations between some of them. Simultaneous usage of strategies was observed in Catholic priests as well (Büssing et al. 2017b), i.e., Interpersonal communication and Self-Care, Interpersonal communication, Self-Care and Spirituality, or Avoidance and Spirituality. Although we cannot verify that specific strategies were preventive buffers, predictor analyses revealed a weak effect of four coping strategies: Avoidance, Private prayer/Meditation/Scripture reading, Voluntary work, and Devotion/Trust in God, which would explain at least 24% of spiritual dryness variance. The Avoidance strategies alone would predict 10% of the spiritual dryness variance. Whether the users of Avoidance strategies are afraid of the ‘spiritual pain’ which might be related to this process, is currently unclear. Most nevertheless do have a deep longing for God, which was significantly higher in persons in leading roles and also in the more effective coping persons. Although longing for God was weakly related to the ability to find ways to cope with spiritual dryness (*r* = .27), it is not a significant predictor of fewer phases of spiritual dryness (*R*^2^ = 0.00).

It is important to underline that phases of spiritual dryness might be inevitable in the life of a religious person. When persons have overcome these phases, several report greater spiritual serenity and depth and are inspired to help others more (Büssing et al. 2017b). In this study, 39% of SDAs were inspired to help others often to regularly (36% not or rarely only), while deeper spiritual clarity and depth were stated by 41% often to regularly (26% not or rarely only). In Catholic priests, 36% were stimulated to help others often to regularly (30% nor or rarely only), and 34% perceived deeper spiritual clarity and depth often to regularly (24% not or rarely only) (Büssing et al. 2017b). Thus, the intention to help after these phases was similar between Catholic priests and SDAs, while spiritual depth after overcoming phases of spiritual dryness was higher in the SDAs. However, among the SDAs, those without any duty in the church had the lowest helping intention and spiritual depth perception compared to those in leading roles or other duties. This no-duty group may require special attention by their pastors, elders and deacons, because they obviously struggle most with phases of spiritual dryness, have lower well-being and less effective coping strategies.

The findings of this study among SDAs suggest that religious trust and meaningful duties in the church (which means to be recognized as an important part of the community) are significant factors to cope with spiritual dryness. Both one´s own spiritual efforts and participation in the church are relevant to endure these phases, to overcome spiritual dryness and to grow as a faithful person. When they are involved in the church and have a duty (‘function’), when they are recognized and needed (in terms of meaning finding), they even more effectively utilize the available coping strategies. A theological substantiation could be the Apostle Peter who supported the idea of a “holy priesthood” of all believers (“chosen people”) to build the church as a living community (1. Peter 2, 5–9). In the pastoral letters of the Apostle Paul (Romans 12, Ephesians 4, 1. Corinthians 12), the church is described as a body that can only be alive and healthy if all believers bring their functions, gifts, and abilities, and when they are “transformed by the renewing of [their] mind” (Romans 12, 2). In this sense, the community (‘church’) could be a ‘training center’ of spiritual growth. However, imagine someone notices that their abilities are not needed. Frustration might be the consequence, lack of participation, and finally inner withdrawal and distancing form God. Thus, pastors, elders and deacons are charged to perceive these tendencies and to support the respective persons.

## Limitations

The sample is probably not representative of all SDAs in Europe. We have focused on SDAs from German language and Francophone countries which share a similar cultural background. It was so far not the intention to compare cultural peculiarities and characteristics which may play a further role in how one utilizes the respective coping strategies. We are also aware that an online survey can be accessed only by persons with an internet access, and further that we might have been unable to invite the ‘passive’ members of the SDA church.

We have no data at what time in their life the participants have experienced spiritual dryness, only how often they did experience them. Also, the intensity of their perceptions was not quantified, and one may assume that in some cases these phases might be ‘mild’, while they can be ‘severe’ in other cases. Nevertheless, here we asked how they were able to cope with these phases, which strategies were used, and which outcomes were observed.

## Conclusion

Phases of spiritual life were perceived by several persons as part of their religious life. Some theologians consider this a necessary experience (Rahner 1993). Nevertheless, the experience of spiritual dryness may be seen as a crossroads either to spiritual growth and a mature relationship with God, or spiritual desolation or even loss of faith (Büssing 2019). In most cases, the SDAs investigated herein found ways to cope with spiritual dryness, and several experienced spiritual depth and helped others all the more after overcoming these phases. It is interesting that the strategies which were found to be most helpful to cope with spiritual dryness are also those which can cause the problems indicating spiritual dryness. Thus, when God is perceived as non-responsive and distant and the prayer life thus becomes difficult, to keep on praying (in terms of a persisting longing for God) and trusting that God is still listening, is an advise which is justified by the strategies which were perceived as helpful by others. The beneficial strategies should be made known to others struggling with perceptions of spiritual dryness. Adequate support means first to acknowledge that these perceptions are not necessarily a matter of failure of ‘weakness of faith’ but a vulnerable process of spiritual development, and second to talk about these experiences at eye level (and not as spiritual experts who pretend to be closer to God). It was striking that particularly persons without a specific duty in the church experienced more often phases of spiritual dryness, had more problems to cope with them, and seem to have less effective strategies. Recognizing them with their abilities and charism could be a first step to helping them stabilize their faith and their commitment in the religious community.
